# Combining biopsy needle and virtual bronchoscopy for tuberculosis‐induced complete bronchial blockage

**DOI:** 10.1002/rcr2.70026

**Published:** 2024-09-16

**Authors:** Yoshio Nakano, Norio Okamoto, Yuri Enomoto, Iwao Gohma, Hiroyuki Hukuda, Yoko Yamamoto, Naoki Ikeda

**Affiliations:** ^1^ Department of Respiratory Medicine Sakai City Medical Center Sakai City Japan; ^2^ Department of Thoracic Surgery Sakai City Medical Center Sakai City Japan

**Keywords:** balloon dilation, complete bronchial obstruction, endobronchial tuberculosis (EBTB), transbronchial aspiration needle, virtual bronchoscopy

## Abstract

Endobronchial tuberculosis (EBTB) presents significant clinical challenges, particularly when complete bronchial obstruction occurs. In this case, a young woman with right main bronchus occlusion due to tuberculosis (TB) was treated using a novel approach. Instead of using a traditional rigid bronchoscope, a flexible approach was adopted. Under precise fluoroscopic guidance, a 21‐gauge transbronchial aspiration needle was used to puncture the obstruction, allowing passage of the guidewire and subsequent balloon dilation. The use of virtual bronchoscopy, developed using computed tomography scans, ensures safe navigation around critical vascular structures. Postoperatively, the patient showed significant symptomatic improvement without complications. This innovative approach not only demonstrates the efficacy and safety of using biopsy needles and virtual bronchoscopy for managing complete bronchial obstructions in EBTB but also opens the door for future innovative solutions in such complex cases.

## Graphical abstract

Our case report details the successful use of a biopsy needle to puncture a completely obstructed bronchus, followed by balloon dilation facilitated by virtual bronchoscopy. This technique, performed under precise fluoroscopic guidance, allowed safe and effective management of the obstruction, leading to significant patient improvement without complications.

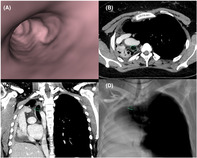

## INTRODUCTION

Tuberculosis (TB) remains a significant global health challenge affecting millions of people worldwide. In 2015, the global incidence of TB was approximately 10.2 million new cases, with a prevalence of approximately 10.1 million cases.^1^

EBTB is a form of TB involving the tracheobronchial tree. Although the exact incidence of EBTB is unknown, it was common before anti‐tuberculosis therapy. Currently, EBTB is reported in 6%–54% of patients with pulmonary TB.^2^

The primary treatment goal for EBTB is to eradicate *Mycobacterium tuberculosis* infection and prevent bronchial stenosis. Treatment typically involves antituberculosis drugs supplemented with bronchoscopic or surgical interventions, as needed. Bronchoscopic interventions, such as bronchial dilatation, stent insertion, laser therapy, cryotherapy, and argon plasma coagulation, are used to restore airway patency. Removable stents are recommended to avoid long‐term complications.^3^

No reported cases have outlined specific management strategies for situations in which a guidewire cannot pass through a completely obstructed trachea. This case addresses the complete occlusion of the right main bronchus caused by EBTB by puncturing the obstruction using a biopsy needle to create an opening for the guidewire. This allowed for the subsequent passage of a balloon catheter, effectively relieving the obstruction. A virtual bronchoscopic image was created using computed tomography (CT)‐identified blood vessel locations. The puncture site was confirmed under fluoroscopic guidance, enabling the procedure to be performed without complications, such as massive bleeding or pneumothorax.

## CASE REPORT

A healthy woman in her 20s was treated for pulmonary TB and EBTB at another hospital from X to X + 183 days using a regimen of 2HREZ/4HR. She began experiencing mild exertional dyspnea, and chest radiography on day X + 366 revealed right lung atelectasis. She was referred to our hospital on day X + 414 for treatment, and bronchoscopic intervention was performed on day X + 431.

The patient's physical characteristics were as follows: height, 154 cm; weight, 69 kg; and BMI 29. Her SpO2 was 97% on room air, and breathing sounds were diminished in the right chest.

**FIGURE 1:**
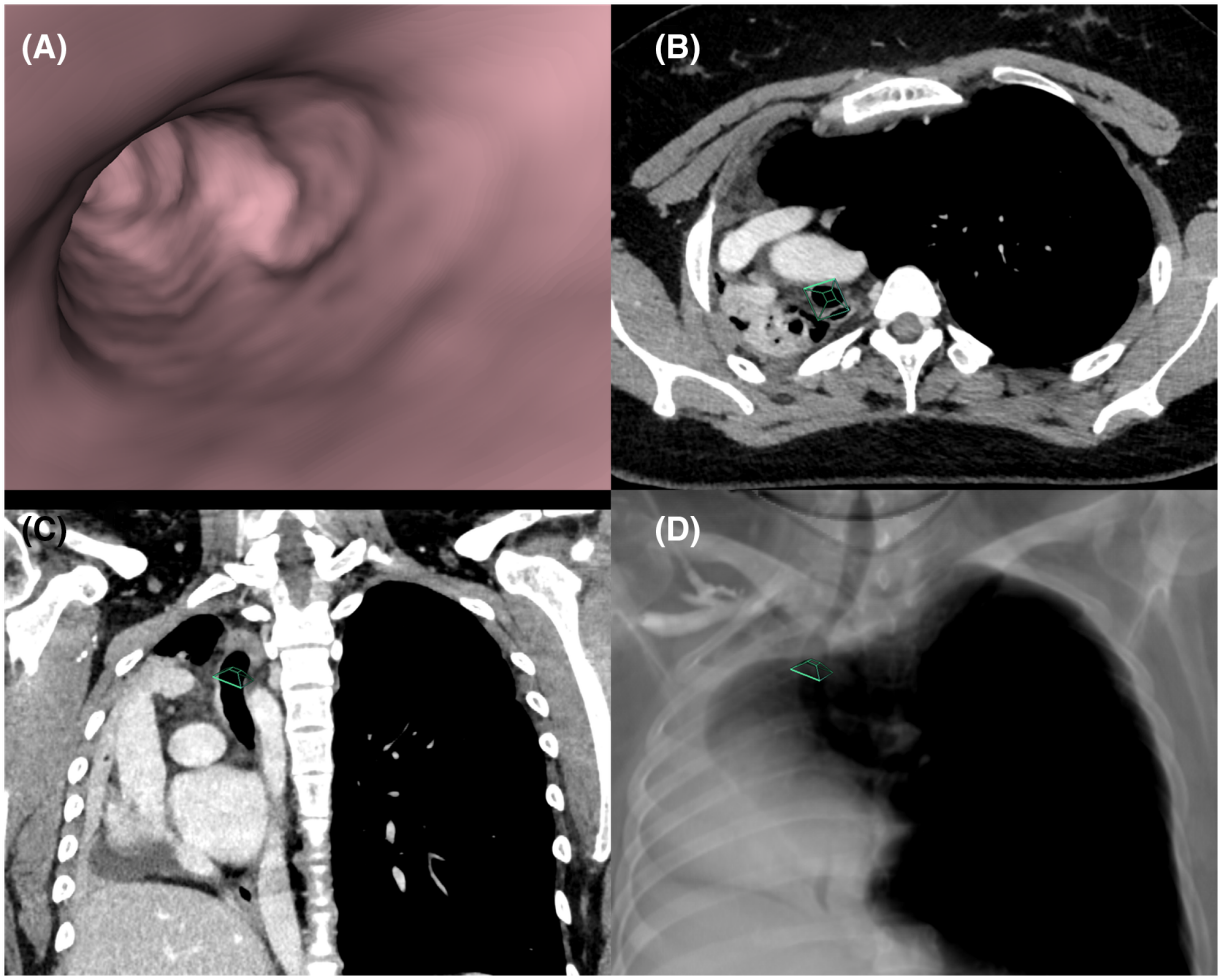
Chest computed tomography (CT) images. (A) Identification of the right main bronchial obstruction site using virtual bronchoscopy. (B) Axial image of the obstruction site. (C) Coronal image of the obstruction site. (D) RaySum (Ray Summation) constructed from CT images.

Preoperative CT imaging, including virtual bronchoscopy, was used to evaluate the relationship between the occluded segment and adjacent vasculature, ensuring a safe puncture direction. CT scan revealed a membranous occlusion at the site of bronchial obstruction. Virtual bronchoscopy played a crucial role in this procedure by providing a clear three‐dimensional view of the bronchial tree, which helped in planning the puncture and avoiding critical vascular structures (Figure 1).

**FIGURE 2:**
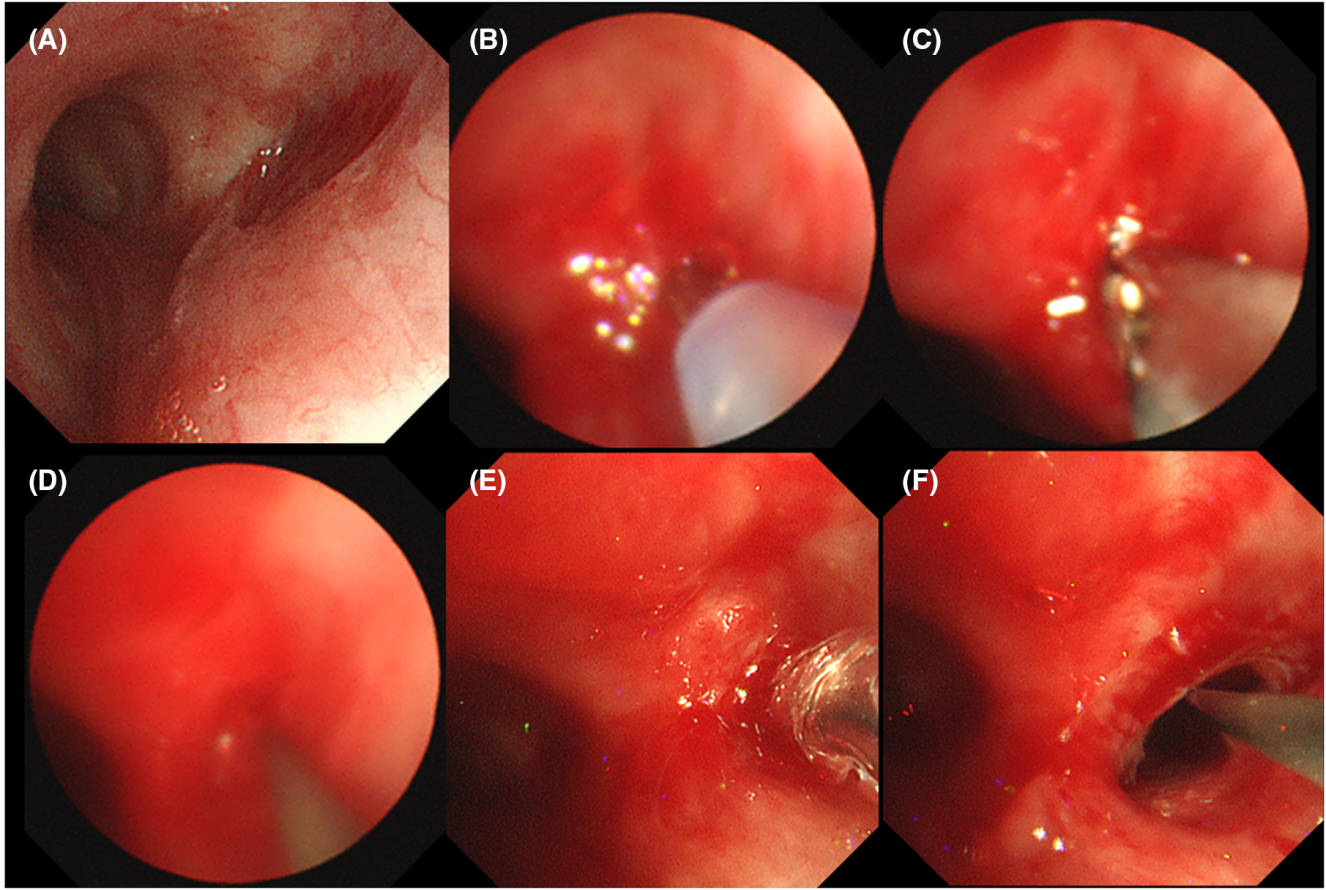
Bronchoscopic images. (A) Complete obstruction of the right main bronchus. (B) Puncture at the right main bronchial obstruction site with a biopsy needle. (C) Passage of biopsy forceps through the hole created by the biopsy needle. (D) Passage of a guidewire through the enlarged hole. (E) Dilation with a balloon catheter. (F) Post‐procedure, successful recanalization of the left main bronchus.

The procedure was performed under general anaesthesia. Initial attempts using a rigid bronchoscope were unsuccessful because of tracheal deviation to the right and central tracheal stenosis, which prevented further advancement of the rigid scope. Subsequently, the rigid scope was removed and tracheal intubation was performed. The procedure was continued by using a flexible bronchoscope. The bronchial stenosis was complete (Figure 2A) and attempts to pass a guidewire through the occlusion were unsuccessful.

**FIGURE 3:**
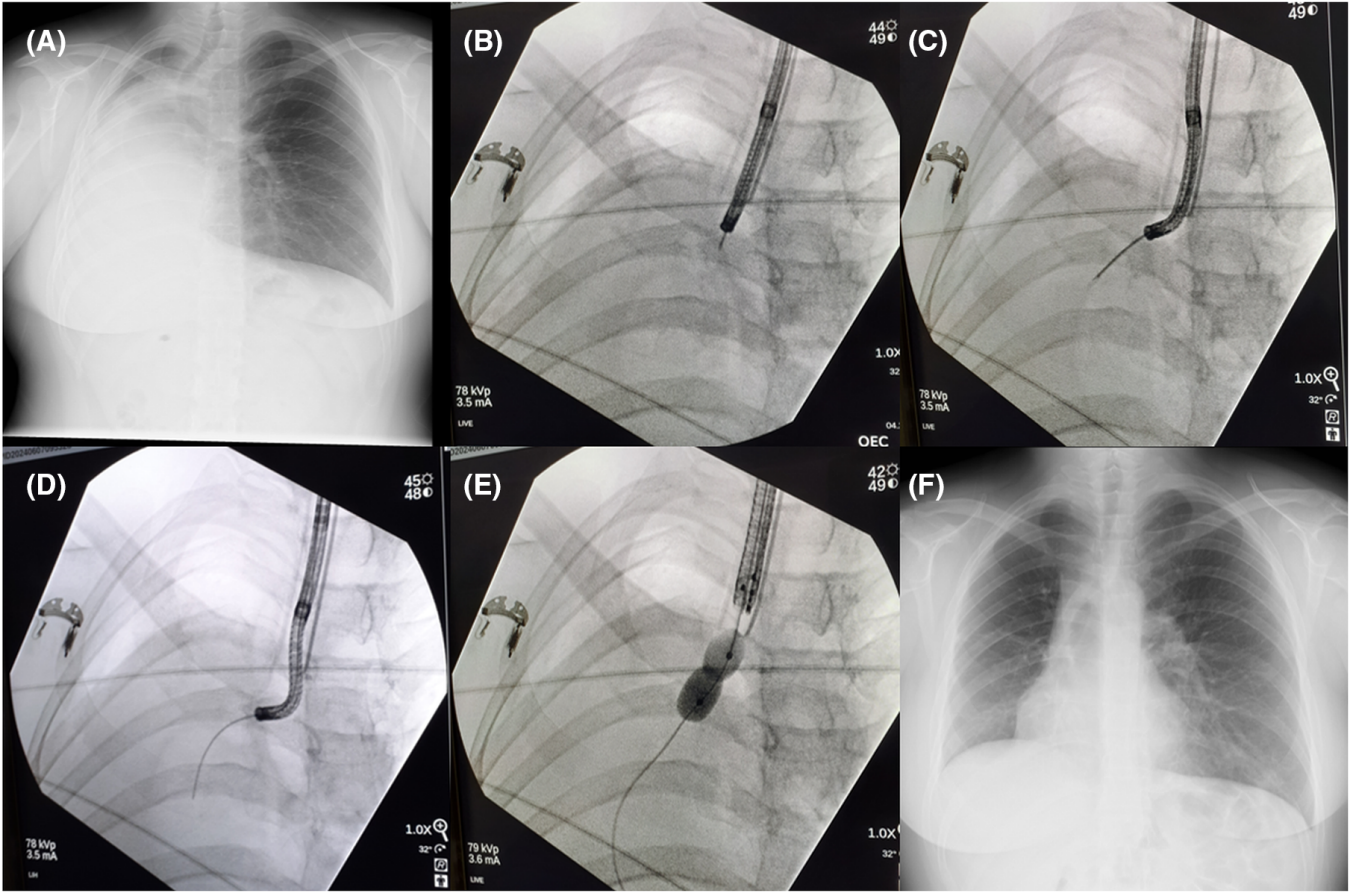
Chest x‐ray images. (A) Preoperative x‐ray showing right atelectasis. (B) Puncture at the right main bronchial obstruction site with a biopsy needle. (C) Passage of biopsy forceps through the hole created by the biopsy needle. (D) Passage of a guidewire through the enlarged hole. (E) Dilation with a balloon catheter. (F) Improvement of atelectasis confirmed in the x‐ray taken 5 days post‐procedure before discharge.

Multiple punctures were performed under fluoroscopic guidance to navigate through the stenosis using a flexible 21‐gauge transbronchial aspiration needle (PeriView FLEX NA‐403D‐2021; Olympus Corporation, Tokyo, Japan) (Figure 2B, Figure 3B). The puncture sites were dilated using biopsy forceps (Figures 2C and 3C). Ultimately, a guidewire was successfully passed through the stenosis (Figures 2D and 3D), allowing a bronchoscope with a diameter of 4 mm to advance through the narrowed segment. The post‐stenotic bronchi in the right main bronchus were confirmed to be patent.

The preoperative chest X‐ray revealed right lung atelectasis (Figure 3A). For stenosis at the entrance of the right main bronchus, a CRE Pulmonary Balloon Dilator (Boston Scientific Corporation, Marlborough, MA, USA) was used. Dilations were performed at 3 atm for 120 s twice and at 8 atm for 120 s twice (Figures 2E and 3E). Initially, the bronchoscope with a 6 mm diameter had difficulty passing through, but eventually, it was able to pass through the dilated segment (Figure 2F). Haemostasis was confirmed and the procedure was concluded.

Postoperatively, the patient's respiratory status and symptoms, including dyspnea and chest discomfort, improved significantly. Bronchoscopy on the fifth postoperative day confirmed no restenosis, and the patient was discharged on the sixth postoperative day with a marked improvement in her quality of life.

## DISCUSSION

This case report demonstrates two key points. First, in the treatment of EBTB with complete obstruction of the right main bronchus, it is possible to puncture and create a hole using a biopsy needle, allowing the guidewire to pass through the obstructed site. This facilitated the introduction of a balloon catheter, which enabled dilation of the obstructed area. Second, by creating a CT‐based virtual bronchoscopy image in advance to confirm the position of blood vessels and using fluoroscopy to verify the puncture site, the procedure was performed safely without major complications such as massive bleeding or pneumothorax, providing reassurance and confidence in the approach.

The biopsy needle proved useful in cases in which the bronchus was completely obstructed by bronchial TB. Similar cases have been reported, in which bronchoscopic balloon dilation was successfully performed to treat almost complete occlusion of the right main bronchus in a 77‐year‐old female patient.^4^ However, specific bronchoscopic approaches for complete occlusion have not been reported. Transbronchial needle aspiration (TBNA) is demonstrated to be an effective and safe method for diagnosing peripheral pulmonary lesions (PPLs).^5^ Using a biopsy needle, a hole was created in the completely obstructed site, allowing the guidewire to pass through. This method is useful even in cases of complete obstruction and can be managed using a flexible bronchoscope.

Additionally, by creating a CT‐based virtual bronchoscopy image in advance to confirm the relationship between the obstructed site and blood vessels, the procedure was performed without complications, such as vascular mis‐puncture or pneumothorax. Preoperative preparation using virtual bronchoscopy is beneficial in patients with complete bronchial obstruction.

## AUTHOR CONTRIBUTIONS

Yoshio Nakano and Norio Okamoto conceived the study, prepared the data, and drafted the manuscript. Yuri Enomoto and Iwao Gohma interpreted the data and revised the manuscript. Hiroyuki Hukuda and Yoko Yamamoto supervised the study and reviewed the manuscript. Naoki Ikeda managed the project and approved the final manuscript. All authors approved the final version of the manuscript.

## CONFLICT OF INTEREST STATEMENT

None declared.

## ETHICS STATEMENT

The authors declare that appropriate written informed consent was obtained for the publication of this manuscript and accompanying images.

## Data Availability

Data sharing not applicable to this article as no datasets were generated or analysed during the current study.
